# Non-grasping en bloc mediastinal lymph node dissection for video-assisted thoracoscopic lung cancer surgery

**DOI:** 10.1186/s12893-015-0025-1

**Published:** 2015-04-08

**Authors:** Chengwu Liu, Qiang Pu, Chenglin Guo, Zhilan Xiao, Jiandong Mei, Lin Ma, Yunke Zhu, Hu Liao, Lunxu Liu

**Affiliations:** Department of Thoracic Surgery, West China Hospital, Sichuan University, No. 37, Guoxue Alley, Chengdu, Sichuan 610041 China

**Keywords:** Mediastinal lymph node dissection (MLND), Video-assisted thoracoscopic surgery (VATS), Lung cancer

## Abstract

**Background:**

This study aims to introduce an optimized method named “non-grasping en bloc mediastinal lymph node dissection (MLND)” through video-assisted thoracoscopic surgery (VATS).

**Methods:**

Between February 2009 and July 2013, 402 patients with clinical stage I non-small cell lung cancer (NSCLC) underwent “non-grasping en bloc MLND” conducted by one surgical team. Target lymph nodes (LNs) were exposed following non-grasping strategy with simple combination of a metal endoscopic suction and an electrocoagulation hook or an ultrasound scalpel. In addition, dissection was performed following a stylized three-dimensional process according to the anatomic features of each station. Clinical and pathological data were prospectively collected and retrospectively reviewed.

**Results:**

The postoperative morbidity and mortality were 17.4% (70/402) and 0.5% (2/402), respectively. The total number of LNs (N1 + N2) was 16.0 ± 5.9 (range of 5–52), while the number of N2 LNs was 9.5 ± 4.0 (range of 3–23). The incidences of postoperative upstaging from N0 to N1 and N2 disease were 7.7% and 12.2%, respectively.

**Conclusions:**

Non-grasping en bloc MLND enables en bloc dissection of mediastinal LNs with comparable morbidity and oncological efficacy while saving troubles of excessive interference of instruments and potential damage to the target LN.

## Background

Accurate lymph node (LN) staging is an important component of the assessment and management of patients with resectable non-small cell lung cancer (NSCLC). Although the role for mediastinal LN sampling (MLNS) versus mediastinal LN dissection (MLND) with regard to improvement in survival of early-stage NSCLC is still controversial, the necessity for LN assessment for staging purpose is undeniable [1-5]. Therefore, current guidelines recommend that systemic mediastinal LN sampling or dissection with at least three N2 stations assessed is mandatory for all patients with resectable NSCLC [3,4,6].

Video-assisted thoracoscopic surgery (VATS) became a viable treatment option for NSCLC with an equivalent oncological resection (lobectomy plus MLND or MLNS) [5,7-11] and equivalent or even superior oncological outcomes [12]. However, MLND through VATS has always been an aporia. Except for technical difficulties caused by limited access ports and narrow operative space, surgeons always face a dilemma that on one hand they should accomplish “completeness” with stronger grip or traction of the target LNs, while on the other hand they should avoid cutting or crushing the target LNs. Recently, some experts depicted their techniques in performing VATS MLND, and it’s not unique that all of them had used strategies to grasp the target LNs [13-15]. The present study has developed different and novel strategies to avoid direct grasping of the target LNs. We proposed the concept of “single-direction VATS lobectomy” [16], accompanied with which we gradually developed a stylized method of MLND named as “non-grasping en bloc MLND”. The aim of this study is to depict our stylized technical tricks and to evaluate its quality and safety.

## Methods

### Patients

Between February 2009 and July 2013, 402 patients with clinical stage I (T1a, 1b, 2a N0M0) NSCLC underwent “VATS lobectomy” plus “non-grasping en bloc MLND” performed by one surgical team in our department. Informed consent was obtained from every patient before the operation. All patients underwent routine systemic function assessments, including blood tests and cardiopulmonary function text preoperatively. The preoperative staging consisted of routine computerized tomography (CT) scanning of the thorax and the upper abdomen, CT scanning or magnetic resonance imaging of the brain, bone scintigraphy, and bronchoscopy. The 18 F-fluorodeoxyglucosepositron emission tomography/CT (18 F-FDG-PET/CT) and/or mediastinoscopy were performed to those subjects with suspicious LN involvement shown on CT scans. Patients with documented positive LNs involvement were excluded from this study. Clinical and pathological data were prospectively collected and retrospectively reviewed for every patient. All LNs harvested were counted by the surgeon himself immediately after the operation and checked by pathologists postoperatively. Informed consent was obtained from each patient. This study was approved by the institutional review board of our hospital.

### Surgical techniques

General anesthesia is administered to each patient through double-lumen endotracheal intubation. Each patient is placed in the appropriate lateral decubitus position. A 1.5 cm observation incision is made in the seventh intercostal space at the midaxillary line for the thoracoscope while two additional utility incisions are placed as follows: a 3 cm main utility incision is made at the anterior axillary line in the third intercostal space for the upper and middle lobes and in the fourth intercostal space for the lower lobes, and a 2 cm assistant utility incision is made in the ninth intercostal space (between the posterior axillary line and subscapular line). The surgeon stands in an anterior position to the patient. We prefer to perform lymphadenectomy after lobectomy, while some experts prefer to begin with lymphadenectomy. Lobectomy is performed following the “single-direction” strategy as in our previous description [16]. Dissection of LNs of stations 10, 11, and 12 is performed along with the lobectomy. The intralobar LNs (stations 13 and 14) were retrieved along with the resected lobe and anatomized by the surgeon himself. As recommended, we routinely dissect stations 2, 4, 7, 8, and 9 for right-side lobectomy and stations 4, 5, 6, 7, 8, and 9 for left-side lobectomy. Dissection of station 3 is performed only for selected patients if there are suspicious LNs found during operation.

It is better to perform video-assisted thoracoscopic MLND with fewer tools because of limited access ports. Keeping this point in mind, we have explored diversified and novel use of an ordinary metal endoscopic suction (MES) with side holes on the tip. Because of its suction capacity, MES can be used to “grasp” the target structure, and then to facilitate exposure by lifting or doing slight side compression of the target structure. This device can play simultaneous roles as peanut, grasper, and suction. Additional grasper or retractor and accompanied mutual interference among instruments can be saved. The MES can immediately suck away the smog or ooze to ensure a clear visual or operating field. In addition, an electrocoagulation hook (EcH), which plays roles as dissector and sealer, is used for precise excision and hemostasis. An ultrasonic scalpel (US), which produces reliable and durable ligation of small lymphatic or blood vessels, plays roles as blunt dissector, sealer, and clamper. A simple combination of the MES and the alternating use of the EcH or US are helpful in maintaining a clear operating field and are effective enough in dissection. During the operation, we avoided grasping the target LNs directly as possible. During a right-side procedure the MES is inserted through the assistant utility incision while the EcH or US was inserted through the main utility incision. However, during a left-side procedure the MES is inserted through the main utility incision while the EcH or US is inserted through the assistant utility incision. Instead of dissecting the target LNs only, we attempt to dissect the total fat pad located among the anatomic landmarks of each station. Adhering to the en bloc strategy, we carry out three-dimensional dissection following specific orders according to different anatomic features of each station. Modular dissection is carried out station by station. Detailed operative techniques are described as follows.

#### 2 R and 4 R

**Figure 1:**
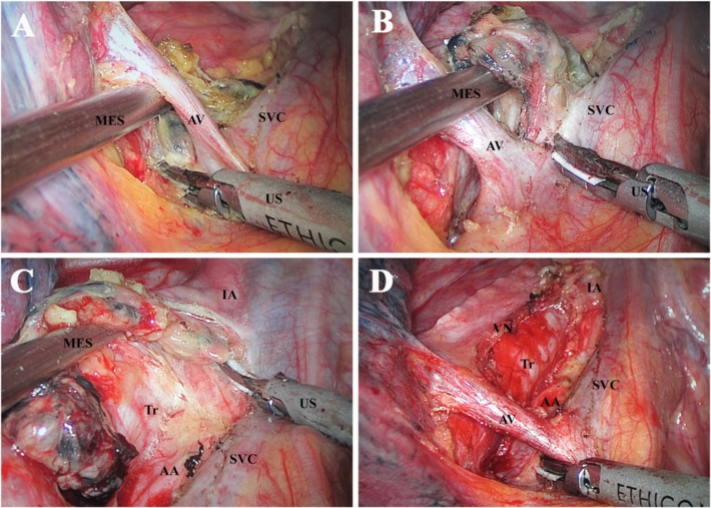
**Dissection of station 2R, 4R. (A)** Initiating the dissection right beneath the azygos vein and hollowing out the block from the interspace surrounded by the arch of azygos vein, superior vena cava, lower trachea, and ascending aorta. **(B)** Flipping the block over the arch of azygos vein and lifting it by the MES. **(C)** Dissecting the block off the superior vena cava, ascending aorta, and trachea sequentially from the cephalad border of the azygos vein to the caudal border of the innominate artery. **(D)** Anatomic landmarks after dissection. *MES* metal endoscopic suction, *US* ultrasonic scalpel, *AV* azygos vein, *SVC* superior vena cava, *Tr* trachea, *AA* ascending aorta, *IA* innominate artery, *VN* vagus nerve.

With retraction of the remnant lung toward the right posterior costophrenic corner, the mediastinal pleura is opened by the EcH along the cephalad and caudal border of the azygos vein, and along the posterior border of the SVC to the caudal border of the innominate artery. With the help of MES, dissection of the block is initiated right beneath the arch of azygos vein by the US. The block is first dissected off the arch of azygos vein, and then hollowed out from the interspace surrounded by the arch of azygos, SVC, lower trachea, and ascending aorta (Figure 1A). Then the lower part of the block is free and will be flipped over the arch of azygos vein and lifted by the MES (Figure 1B). Next, the block is slightly pushed aside or lifted by the MES and dissected off the posterior border of the SVC, the lateral border of the ascending aorta, and the anterior border of the trachea sequentially from the cephalad border of the azygos vein to the caudal border of the innominate artery (Figure 1C). “Grasped” by the MES, the block will be dissected longitudinally and anterior to the vagus nerve en bloc (Figure 1D). Small venous or lymphatic vessels draining the mediastinal fat pad are ligated and cut by the US. At the apex, the right recurrent laryngeal nerve should be kept in mind and protected from thermal or mechanical injury with no necessity to expose it desperately.

#### 7 R

**Figure 2:**
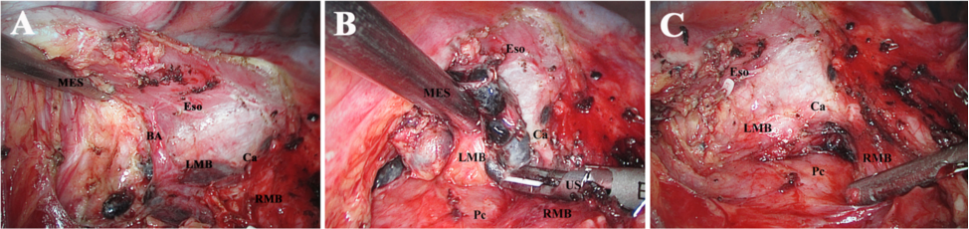
**Dissection of station 7 from the right side. (A)** Dissecting the block off the esophagus. **(B)** Dissecting the block off the carina. **(C)** Anatomic landmarks after dissection. *MES* metal endoscopic suction, *Eso* esophagus, *RMB* right main-stem bronchus, *BA* bronchial artery, *LMB* left main-stem bronchus, *Ca* carina, *Pc* pericardium.

The remnant lung is retracted toward the right anterior costophrenic corner to expose the posterior mediastinum and to increase the angle between the right and left main-stem bronchi. The mediastinal pleura is opened by the EcH posterior to the right main-stem bronchus and anterior to the esophagus, from the inferior ligament, up to the arch of azygos vein. The subcarinal block is first dissected off the esophagus until the left main-stem bronchus and the carina are identified (Figure 2A). During this process, the esophagus is pushed aside with the MES, and the block is detached from the esophagus mainly by EcH. In addition, a small bronchial artery arising from the aorta and entering into the right lung that is frequently present can be ligated with hemoclips and then cut by the US (Figure 2A). Retracted by the MES with certain tension, the block is then dissected off the pericardium and along the posterointernal border of the right main-stem bronchus to the level of carina by the US. Until here, the block has been dissociated three-dimensionally. At last, the block can be dissected off the carina and retrieved en bloc (Figure 2B and C), and when doing so, small feeding vessels entering into the subcarinal LNs from the region of the carina should be carefully dissected by the US to avoid bothersome bleeding. Meticulousness should be maintained to avoid injury to the membraneous portion of the right and left main-stem bronchi.

#### 5 and 6

**Figure 3:**
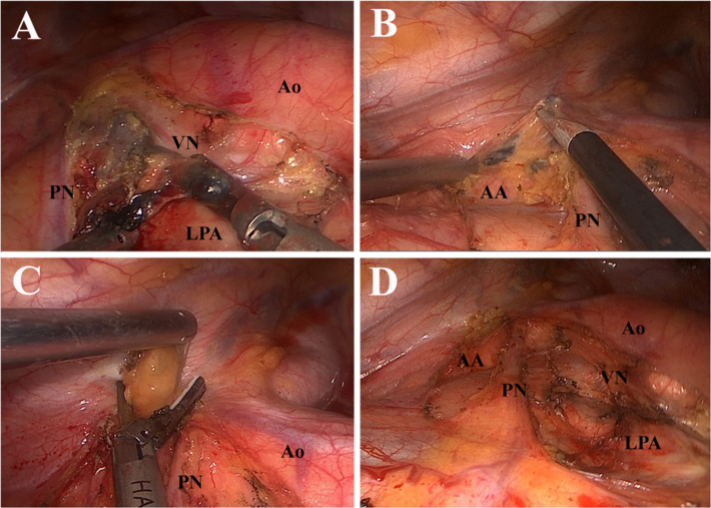
**Dissection of station 5 and 6. (A)** Dissecting the block of station 5 anterior to the vagus nerve and posterior to the phrenic nerve. **(B)** Opening the mediastinal pleura anterior to the phrenic nerve. **(C)** Grasped by the MES the block of station 6 is dissected by the US leaving the phrenic nerve hung free. **(D)** Anatomic landmarks after dissection. *PN* phrenic nerve, *VN* vagus nerve, *Ao* aorta, *LPA* left pulmonary artery, *AA* ascending aorta.

With retraction of the remnant lung toward the left posterior costophrenic corner, the mediastinal pleura is opened by the EcH posterior to the phrenic nerve and anterior to the vagus nerve, from the upper rim of the left main pulmonary artery to the aortic arch. The station 5 LN block is firstly dissected off the left main pulmonary artery by the EcH. Pushed aside or “grasped” by the MES, the block is then dissected by the US anterior to the vagus nerve and posterior to the phrenic nerve (Figure 3A). The station 6 LN block is commonly located between the phrenic nerve and the ascending aorta. We usually open the mediastinal pleura anterior to the phrenic nerve with the EcH (Figure 3B) and dissect the block with the US leaving the phrenic nerve hung free (Figure 3C and D). During the operation, the surgeon must keep in mind not to injure the phrenic and the vagus nerves.

#### 4 L

**Figure 4:**
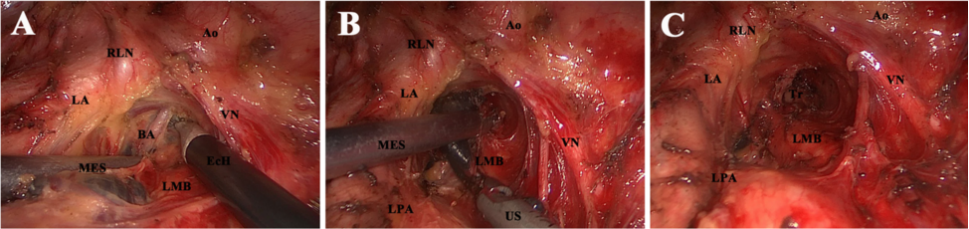
**Dissection of station 4 L. (A)** Dissecting the block off the arch of aorta. **(B)** Dissecting the block off the left pulmonary artery and the left main-stem bronchus. **(C)** Anatomic landmarks after dissection. *MES* metal endoscopic suction, *EcH* electrocoagulation hook, *RLN* recurrent laryngeal nerve, *LA* ligamentum arteriosum, *VN* vagus nerve, *Ao* aorta, *BA* bronchial artery, *LMB* left main-stem bronchus, *LPA* left pulmonary artery, *US* ultrasonic scalpel, *Tr* trachea.

With retraction of the remnant lung to the anterior costophrenic corner, the pleural area between the ligamentum arteriosum, left main pulmonary artery, vagus nerve, and left main-stem bronchus is opened by the EcH. The block is pressed downward by the MES, and is first dissociated from the inferior border of the aortic arch mainly by the EcH (Figure 4A). A bronchial artery that is frequently present here can be ligated (Figure 4A). Then, the block is dissected off the left main pulmonary artery and along the left main-stem bronchus to the trachea by the US (Figure 4B). Finally, the block is hollowed out from the interspace between the aortic arch, the left main pulmonary artery, and the left main-stem bronchus (Figure 4C). The left recurrent laryngeal nerve must be identified and meticulously protected, but with no need to anatomize it desperately.

#### 7 L

**Figure 5:**
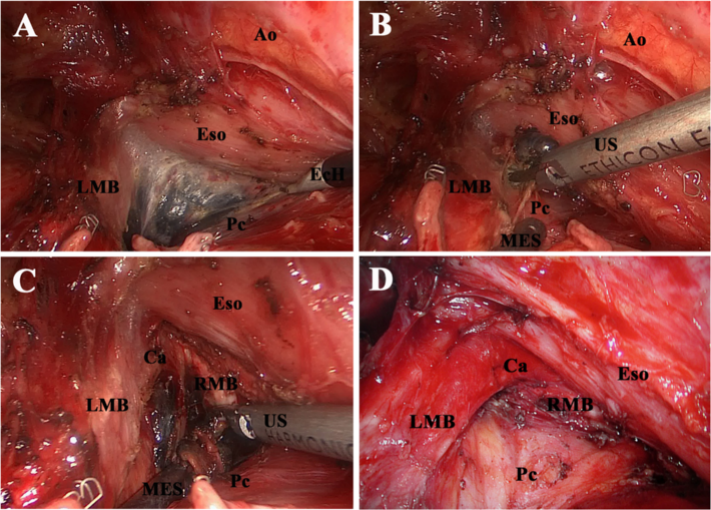
**Dissection of station 7 from the left side. (A)** Dissecting the block off the esophagus. **(B)** Dissecting the block off the pericardium and the left main-stem bronchus. **(C)** Dissecting the block off the right main-stem bronchus and the carina. **(D)** Anatomic landmarks after dissection. *Ao* aorta, *Eso* esophagus, *LMB* left main-stem bronchus, *Pc* pericardium, *Ca* carina, *MES* metal endoscopic suction, *US* ultrasonic scalpel, *RMB* right main-stem bronchus.

Dissection of the subcarinal nodes from the left side is more difficult and time-consuming than that from the right side. With retraction of the remnant lung forward, the pleura area between the left main-stem bronchus, pericardium, and esophagus is opened. Similar to that performed on the right side, the subcarinal fat pad is first dissected off the esophagus by the EcH until the right main-stem bronchus and the carina are identified (Figure 5A). Then, the block is dissected off the pericardium and posterointernal border of the left main-stem bronchus by the US (Figure 5B). After that, the block is dissected off the right main-stem bronchus and the carina and retrieved en bloc by the US (Figure 5C and D). The small feeding vessels, which commonly enter into the LNs from the anterior border of the trachea at the level of the carina, must be identified, and clipped or ligated to avoid bleeding. The membranous portion of the left and right main-stem bronchi should not be injured.

#### 3, 8, and 9

A great variation might exist in the number and consistency of station 3 (prevascular and retrotracheal LNs), station 8 (paraesophageal LNs), and station 9 (LNs embedded in pulmonary ligament). Nodes can be completely absent. We retrieve station 3 LNs only when there are obvious prevascular and/or retrotracheal lymphadenectasis. We retrieve station 8 LNs, if there would be any, when we perform dissection of station 7 LNs. Dissection of station 9 LNs is accomplished when we cut off the inferior pulmonary ligament.

## Results

There were 172 female and 230 male patients. The mean age was 61.1 ± 10.5 years (range of 25–86 years). Tables 1 and 2 summarize the clinical and pathological data. The incidence of postoperative complications was 17.4% (70/402). The postoperative mortality was 0.5% (2/402). One patient died from respiratory failure caused by postoperative pneumonia and the other one died from postoperative bronchopleural fistula. The total number of LNs (N1 + N2) was 16.0 ± 5.9 (range of 5–52), and the number of N2 LNs was 9.5 ± 4.0 (range of 3–23). The incidences of postoperative upstaging from N0 to N1 and N2 disease were 7.7% (31/402) and 12.2% (49/402), respectively. Table 3 shows the number of LNs harvested from each N2 station.

**Table 1:** **Clinical and pathological data**

**Variant**	**N (n = 402)**
Procedure	
Left upper lobectomy	96 (23.9%)
Left lower lobectomy	67 (16.7%)
Right upper lobectomy	126 (31.3%)
Right middle lobectomy	35 (8.7%)
Right lower lobectomy	66 (16.4%)
Right upper and middle lobectomy	3 (0.7%)
Right middle and lower lobectomy	9 (2.3%)
Pathology	
Adenocarcinoma	306 (76.1%)
Squamous carcinoma	78 (19.4%)
Adenosquamous carcinoma	12 (3.0%)
Carcinoid	2 (0.5%)
Others^a^	4 (1.0%)
Pathological stage	
Ia	115 (28.6%)
Ib	207 (51.5%)
IIa	31 (7.7%)
IIIa	49 (12.2%)
Complications	
Pneumonia	26 (6.5%)
Air leakage >7 days^b^	12 (3.0%)
Drainage >7 days^c^	7 (1.7%)
Arrhythmia	5 (1.3%)
Asthma	3 (0.7%)
Chylothorax	3 (0.7%)
Recurrent laryngeal nerve paralysis	3 (0.7%)
Pneumonia + Drainage >7 days	2 (0.5%)
Pulmonary embolism	2 (0.5%)
Postoperative hemorrhage	2 (0.5%)
Others^d^	5 (1.3%)

^a^Each case of large cell neuroendocrine carcinoma, clear-cell carcinoma, lymphoepitheloid carcinoma, and compound carcinoma (small cell and squamous cell); ^b^Prolonged air leakage accompanied with or without persisted drainage; ^c^Prolonged drainage only attributed to large amount of daily drainage; ^d^Each case of mental symptom, deep venous thrombosis, wound infection, bronchopleural fistula, and hepatic dysfunction.

**Table 2:** **Postoperative parameters**

**Variant**	**Median/Mean ± SD**	**Range**
Operative time (minutes)	139.6 ± 37.8	70–240
Blood loss (ml)	80.8 ± 99.0	10–700
Duration of postoperative drainage (d)	3.6 ± 2.3	1–23
Amount of postoperative drainage (ml)	699.8 ± 584.3	50–4700
Length of postoperative hospital stay (d)	7.5 ± 3.2	3–26

**Table 3:** **Lymph nodes harvested from each station (N2)**

**Stations (N2)**	**No. patients (%)**	**No. lymph nodes**		
		**Mean ± SD**	**Median**	**Range**
Right side	239			
2	217 (90.8)	1.8 ± 0.1	2	1-5
3	111 (46.4)	1.6 ± 0.1	1	1-4
4	239 (100)	2.0 ± 0.1	2	1-5
7	239 (100)	3.5 ± 0.1	3	1-7
8	105 (43.9)	1.3 ± 0.0	1	1-3
9	214 (89.5)	1.4 ± 0.0	1	1-3
Left side	163			
4	118 (72.4)	1.9 ± 1.0	2	1-5
5	157 (96.3)	1.8 ± 1.0	2	1-5
6	146 (89.6)	1.7 ± 1.0	2	1-4
7	163 (100)	3.1 ± 1.3	3	1-7
8	46 (28.2)	1.2 ± 0.5	1	1-3
9	127 (77.9)	1.4 ± 0.6	1	1-4

## Discussion

Although MLND as associated with improved survival of early-stage NSCLC remains controversial, we still advocate the method in our practice. We think that with complete removal of all resectable LNs, detection of micrometastasis or skip lesions, and the proportion of complete R0 resections are increased, which lead to more accurate tumor staging and reduced local recurrence [17-20]. Although video-assisted thoracoscopic MLND is commonly practiced, there may be concerns regarding oncological effectiveness based on the completeness of MLND [21]. Several previous studies demonstrated the feasibility and efficacy of MLND through VATS [5,7-11]. Some experts also depicted their techniques in performing thoracoscopic MLND using traditional “grasping” technique [13-15]. We designed this retrospective study to share our experience in optimizing surgical techniques and to present our outcomes of non-grasping VATS MLND.

The advantages of VATS, such as magnified view and modified visualization, improve the ability to recognize normal structures and to identify anomalous conditions, minimizing the risk of complications during nodal dissection. On the other hand, the disadvantages include two-dimensional view only and limited mobility of surgical devices because of small access ports. Therefore, alternating introduction and removal of different instruments should be avoided as possible. In addition, fragile as LN is, direct grip should be avoided as much as possible in terms of avoiding cutting or crushing the target LN, which may cause dissemination of cancer cells to the pleural or mediastinal space if the node is involved [9]. In our experience, we gradually developed the method of “non-grasping en bloc MLND” that incorporates several important features compared with traditional grasping technique. These features include, first, diversified use of the MES saves trouble of alternating instruments and provides more convenience for the surgeon. Moreover, the combination of the suction and hemostatic device (EcH or US) provides a clear operating field. Second, the non-grasping strategy avoids damage to LNs, and this meets the principles of surgical oncology. Third, en bloc dissection of the bounded fat block makes sure that there is no LN missed. Fourth, three-dimensional anatomization following specific orders according to different anatomic features makes MLND more vivid and concise. Fifth, even for cases with “fixated” mediastinal LNs, the technique of “non-grasping” does well during dissection, and we think that the usage of grasping technique will not help much in dissection but causing troublesome bleeding or damages to the LNs.

When evaluating the feasibility of the video-assisted thoracoscopic MLND, two significant concerns arise, namely, efficacy and safety. The best way to elucidate the completeness is that there would be neither a node nor fat tissue around each station to be found after dissection. We think that skeletonization of the anatomic region is mandatory to each station with clearly exhibited anatomic landmarks. The cutting or crushing of LN may lead to a problem in counting the number of the retrieved LNs as mentioned by Watanabe [9]. In our practice, we think that the non-grasping and en bloc strategy may decrease the probability of cutting or crushing LNs and facilitate to precisely count the number of them. We anatomized the dissected block of each station to count the number of LNs immediately after the operation. The anatomized block would be checked independently by pathologists after all specimens were sent to them.

In this study, we obtained comparable results with other studies [5,11]. The number of N2 LNs and total LNs (N1 + N2) were 9.5 ± 4.0 and 16.0 ± 5.9, respectively. In addition to the extent of MLND, the incidence of postoperative upstaging is also a significant parameter for evaluation. Previous studies found comparable results for VATS vs. open MLND. In these studies, the incidence of upstaging from N0 to N2 disease was found to range from 1.3% to 2.3% for MLND through VATS [7,11,22]. And Amer et al. reported much higher rate of nodal upstaging (16.6%) even though rigorous preoperative staging protocol including PET/CT was performed to each patient [15]. In our study, the incidence of upstaging from N0 to N2 disease was 12.2%. We believe that our persistence in en bloc dissection might have contributed to find more unexpected N2 disease.

Studies so far have demonstrated comparable postoperative mortality and morbidity of MLND by VATS vs. open lobectomy, which indicates that MLND by VATS is a safe procedure [23]. However, when pursuing oncological completeness, we have encountered three cases of hoarseness with unilateral vocal cord palsy (1 case: right side; 2 cases: left side), which might be attributed to thermal or mechanical injury to the recurrent laryngeal nerve caused by the EcH or US. Therefore, we suggest that there is no need to anatomize the recurrent laryngeal nerve desperately when performing MLND. Moreover, there were three cases of postoperative chylothorax, which had been treated faultlessly, in the early days of our practice. We had not realized that this problem might be due to the thoracic duct injury other than small lymphatic leakage until we encountered two cases of chyle leakage when dissecting station 4 R LNs. For these two cases, we ligated the thoracic duct just above the diaphragm resulting in no postoperative chylothorax. Therefore, when dissecting station 4 (R or L) LNs, we should keep in mind that the vulnerable thoracic duct is crossing to the left just behind the ascending aorta. In short, we argue that only with clear anatomic map in mind and meticulous operation could we decrease complications.

During the early stages of our practice in video-assisted thoracoscopic surgery for lung cancer, we had explored different techniques trying to make MLND more convenient and easier. At last, we developed this non-grasping en bloc technique and found it to be more comfortable for us to better perform VATS MLND. However, the techniques used during the exploring stages were diversified. It was hard for us to compare the non-grasping technique with the former techniques. And this is definitely a limitation of our study. Most thoracic surgeons think that the cutting and crushing of LN may cause dissemination of cancer cells to the pleural or mediastinal space if the node is involved [9]. We performed the non-grasping technique abiding by principles of surgical oncology. There were seldom cut or crushed LNs in our practice, and this was also the reason that the total number seemed to be less than that reported in other studies. Although we failed to evaluate the incidence of cut or crushed LNs, which was also a limitation of our study, we did believe that the non-grasping and en bloc strategy might contribute to reduce the incidence of cut or crushed LNs. Moreover, our preoperative staging protocol was not so rigorous due to economic issues when surgical indication was deemed. Preoperative invasive staging protocol (i.e., mediastinoscopy) was used mainly in patients with multiple or bulky mediastinal LNs. For cases with resectable mediastinal LNs, preoperative invasive staging protocol was not critically applied, and this was a very important limitation of our study. Therefore, we routinely performed complete MLND rather than MLNS for every patient in order to accomplish adequate evaluation of mediastinal LNs. And this might be the main reason for a high rate of postoperative nodal upstaging. We’d like to improve our preoperative staging protocol in the future.

## Conclusions

In conclusion, video-assisted thoracoscopic MLND was equivalent to a comparable dissection through a thoracotomy in the hands of an experienced thoracoscopic surgeon with regard to both morbidity and oncological efficacy [24]. The method of “non-grasping en bloc MLND”, which enables en bloc dissection of mediastinal LNs while saving trouble of excessive interference of instruments and potential damage to the target LN, is safe, concise, and effective with a promising application prospect.

## Abbreviations

MLND: Mediastinal lymph node dissection
VATS: Video-assisted thoracoscopic surgery
NSCLC: Non-small cell lung cancer
LN: Lymph node
MLNS: Mediastinal LN sampling
CT: Computerized tomography
18 F-FDG-PET/CT: 18 F-fluorodeoxyglucosepositron emission tomography/CT
MES: Metal endoscopic suction
EcH: Electrocoagulation hook
US: Ultrasonic scalpel
SVC: Superior vena cava
